# Child feeding and stunting prevalence in left-behind children: a descriptive analysis of data from a central and western Chinese population

**DOI:** 10.1007/s00038-016-0844-6

**Published:** 2016-06-18

**Authors:** Lu Ban, Sufang Guo, Robert W. Scherpbier, Xiaoli Wang, Hong Zhou, Laila J. Tata

**Affiliations:** 1 University of Nottingham, Nottingham, UK; 2 UNICEF China, Beijing, China; 3 Peking University, Beijing, China

**Keywords:** Stunting, Nutritional status, Feeding practice, Rural-to-urban internal migration, Left-behind children, Guardianship

## Abstract

**Objectives:**

To examine the effect of parental rural-to-urban internal migration on nutritional status of left-behind children and how this is related to guardianship.

**Methods:**

We used UNICEF China’s maternal and child health survey data to investigate stunting prevalence and feeding practices in children left behind by rural-to-urban internal migrant parents. We also assessed the effects of primary guardianship which is related closely with parental migration.

**Results:**

Of 6136 children aged 0–3 years, over one-third was left behind by one or both parents. About 13 % were left behind by mothers, leaving guardianship primarily to grandmothers. Left-behind status was not associated with stunting, yet children who were cared for primarily by their fathers had a 32 % increase of stunting compared to children cared for by the mothers [adjusted odds ratio (aOR) = 1.32; 95 % confidence interval = 1.04–1.67]. Children with migrant mothers were less likely to receive age-appropriate breastfeeding (aOR = 0.04;0.02–0.10) and a minimum acceptable diet (aOR = 0.56;0.39–0.79) compared with non-left-behind children.

**Conclusions:**

Guardian’s feeding behaviours varied, and was inappropriate for both children affected and not affected by parent’s rural-to-urban internal migration. Community-based infant and young child feeding counselling and support should be provided to all caregivers.

**Electronic supplementary material:**

The online version of this article (doi:10.1007/s00038-016-0844-6) contains supplementary material, which is available to authorized users.

## Introduction

Internal migration for work is a well-recognized and growing issue in China (Guo 2009). The temporary working population nationwide has reached to 200 million in 2011 with over 30 % increase from the previous year.(National Bureau of Statistics of China 2012) The number of female migrants has also increased and about half of the migrants were women in 2000 (Zai Liang 2004). Most internal migrant workers are from rural areas with disadvantaged socioeconomic circumstances, and financial constraint and restrictions on migrants’ access to education and health welfare in working destinations have led to a great number of split families with children being left behind by one or both of their migrant parents for indefinite periods (Jingzhong and Lu 2011). Absence of one or both parents, particularly the mother, may adversely impact the quality of care, love and attention that children receive (Skrbiš 2008; Ma 2010). A recent systematic review of studies on left-behind children in China showed that left-behind children had lower self-concept and more mental health problems than children in the general population (Wang et al. 2014). Children left behind by one or both parents are often primarily cared by non-parent guardians, such as grandparents. Previous research has suggested that such children may experience more neglect (Gu et al. 2011; Zhong et al. 2012), receive less or inadequate care,(Gao et al. 2010) and have an increased risk of anaemia (Hipgrave et al. 2014) and stunting (He et al. 2007; Cui et al. 2008; Chen et al. 2011) compared to non-left-behind children. However, the impact of split families and non-mother guardianship on children’s nutrition and health status has been less studied in children of very young age in which stunting is mostly formed. In addition, evidence on the association of stunting with child’s left-behind status is inconsistent probably due to confounding by other important risk factors for stunting such as carer’s education level and household income. For example, a recent study from Southeast Asia found that the risk of stunting was not associated with parental absence but was mainly attributable to low carer’s education level (Graham and Jordan 2013). Meanwhile, due to the additional financial support provided by the economic migrant patents, left-behind children may have improved living conditions and nutrition (Graham and Jordan 2013).

We therefore, used a large population-based survey of children from birth to age 3 years across 12 western and central provinces in China to (1) examine the effects of maternal and paternal rural-to-urban internal migration on key nutrition and health indicators of child health defined by the World Health Organization (WHO) (World Health Organization 2008) and United Nations Children’s Fund (UNICEF) (United Nations Children’s Fund 2009) and (2) assess the association of stunting with left-behind status taking into account the impact of guardianship.

## Methods

### Data source and study population

We used two sets of cross-sectional survey data collected in 2010 and 2011, as part of a continuing UNICEF integrated maternal and child health project in rural areas of China. Nineteen rural counties (seven from the 2010 survey and 12 from the 2011 survey) with deprived socioeconomic status and poor maternal and child health were selected by staff at the UNICEF and the Chinese Ministry of Health from 12 central and western provinces (Guansu, Guizhou, Qinghai, Sichuan, Jiangxi, Chongqing, Guangxi, Shaanxi, Shanxi, Tibert, Xinjiang and Inner Mongolia). The survey was conducted by Peking University School of Public Health working with local health authorities. A multi-stage sampling technique was used to select townships and villages in each county. The youngest child (less than 5 years old in the 2010 survey and less than 3 years old in the 2011 survey) was selected from each family. Detailed descriptions of the survey methods have been published previously.(Guo et al. 2013) For this study, only children under 3-years old were included, of which 2838 children (53.1 % are male) from 2010 survey and 3298 (54.5 % are male) from 2011 survey. This study was approved by Ethical Committee of Peking University Health Centre.

Left-behind children were defined as those with one or both parents who had left home to work elsewhere at the time of survey and who had left the main responsibility of the child care to the other parent or other relatives. Children were categorised into two groups of left-behind status—fathers migrated only, mothers migrated (with or without a migrant father)—and a reference group of children with neither parent migrated. All groups were mutually exclusive. As guardianship is correspondingly important, children were also categorised into four groups: children whose primary guardian was the mother (reference group), father, grandparent (including grandmother and grandfather) or another relative (mainly aunt or sister). Children whose parents divorced or died (representing only 2 % of the sample) were excluded from the study.

For each child, anthropometric measurements (including height/length and weight) and detailed information on socio-demographic factors, breastfeeding and child complementary feeding practices were obtained during face-to-face interviews with children’s main guardians using standardized questionnaires adapted from WHO indicators for assessing infant and young child feeding practices (World Health Organization 2008) and UNICEF Multiple Indicator Cluster Survey (United Nations Children’s Fund 2009).

### Defining outcomes and covariates

Stunting was the main outcome and measured using length/height-for-age *Z* scores calculated using the WHO 2006 Child Growth Standard (World Health Organization 2006) and children with stunting were defined as those with length/height-for-age *Z* score <-2 standard deviation. Based on WHO and UNICEF guidelines, (World Health Organization 2008; United Nations Children’s Fund 2009) indicators for assessing child breastfeeding (including ever breastfed, length of breastfeeding, early initiation of breastfeeding and age-appropriate breastfeeding) were extracted for all children, and indicators for assessing child complementary feeding (including minimum dietary diversity, minimum meal frequency, minimum acceptable diet and intake of iron-rich/fortified foods) were available for children aged 6–35 months. We also extracted information on milk feeding, defined as use of liquid milk products such as infant formula, cow’s milk or other animals’ milk at the time of survey, for both breastfed and non-breastfed children. Recordings on basic socio-demographic factors, including the child’s age (defined as 0–5 months, 6–11 months, 12–23 months and 24–35 months), sex (as male and female), whether the child had one or more elder siblings, ethnicity (categorised as Han and non-Han ethnicity), household wealth (defined as the number of household electrical appliances and categorised as 0–2, 3 and 4) and the main guardian’s education level (categorised as illiteracy, primary school, junior high school, and senior high school and above) were also extracted as these could be potential confounders indicated in previous studies. (He et al. 2007; Cui et al. 2008; Chen et al. 2011; Graham and Jordan 2013; Hipgrave et al. 2014).

### Statistical analyses

We examined basic socio-demographic characteristics and guardianship for left-behind and non-left-behind children separately. We calculated the numbers and percentages of children with stunting and different feeding practices by left-behind status and the *p* values for each group of children with different left-behind status compared to the group of non-left-behind children using Chi-square tests and student’s *t* tests. To assess the associations of stunting and different feeding practices with different left-behind status, we used unconditional multivariable logistic regression to calculate odds ratios [with 95 % confidence intervals (CI)] for dichotomous outcomes, and multivariable linear regression to calculate *β* coefficients (with 95 % CIs) for the continuous outcome (length of breastfeeding). We adjusted the results with child’s age, gender, ethnicity, whether the child had elder siblings, guardian’s education attainment, number of household electrical appliances and year of survey. We also repeated the analyses to examine the associations of stunting and different feeding practices with guardianship since guardianship could be an important factor when examining the health of left-behind children. In addition, using multivariable logistic regression we calculated odds ratios with 95 % CIs for stunting associated with both left-behind status and guardianship included in the same model. We also included in the model child’s age, gender, ethnicity, whether the child had elder siblings, guardian’s education attainment, number of household electrical appliances, length of breastfeeding (categorised as 0–5, 6–11, 12–35 months) and milk feeding which were also potentially associated with stunting (Shrimpton and Kachondham 2003). Since the questionnaires used for measuring child complementary feeding practices were only for children aged 6 months and above, (World Health Organization 2008; United Nations Children’s Fund 2009) we repeated the analysis and included indicators for child complementary feeding in a sub-group of children aged 6–35 months.

Since our initial calculation of weighting using population adjustment (Yansaneh 2003) made little difference between the un-weighted and weighted proportions of baseline socio-demographic factors (Table S1), we present results from un-weighted data with adjustment for basic socio-demographic factors including age. In our models, we applied a cluster correction on the identification code for provinces to take into account any potential clustering effects within the same province. Besides the basic socio-demographic factors, we additionally adjusted for the year of survey to account for any potential differences between the two surveys. Missing data on guardian’s education level were treated as separate categories and included in the data analyses. A *p* value less than 0.05 was defined as statistically significant. All data analyses were conducted using STATA SE 11 (College Station, Texas, USA).

## Results

There were 6136 children, 26.7 % of children with father migrated only and an additional 12.8 % with mother migrated (with or without a migrant father). The prevalence of stunting was 16.4 % in non-left-behind children, 15.1 % in children whose fathers had migrated only and 16.6 % in children whose mothers had migrated (Table 1). After adjustment for child’s age, gender, ethnicity, whether the child had elder siblings, guardian’s education attainment, the number of household electrical appliances and year of survey, the odds of stunting was similar in children with neither parent migrated and children with only father migrated (Table 2). However, compared with children with neither parent migrated children whose mothers had migrated had a slightly lower odds of stunting [adjusted OR (aOR) = 0.78, 95 % CI 0.65–0.95]. These children were less likely to be ever breastfed (aOR = 0.30, 95 % CI 0.17–0.52) and had shorter breastfeeding periods [*β* coefficient = −3.77, 95 % CI −5.01 to −2.3) (Table 2). These children were also less likely to have minimum meal frequency and minimum acceptable diet but were more likely to receive non-breast milk feeding (Table 2).

**Table 1:** Socio-demographic characteristics, stunting and feeding practices in children with and without migrated parent (*N* = 6,136, China, 2010–2011)

Characteristics of children	Neither parent migrated	Father migrated only	Mother with/without father migrated
	*n* = 3708	*n* = 1640	*n* = 788
	*n* (%)	*n* (%)	*n* (%)
Age of child, months			
0–5	424 (11.4)	192 (11.7)	15 (1.9)
6–11	913 (24.6)	492 (30.0)	111 (14.1)
12–23	1408 (38.0)	609 (37.1)	345 (43.8)
24–35	963 (26.0)	347 (21.2)^a^	317 (40.2)^a^
Child’s gender, male	2015 (54.3)	864 (52.7)	427 (54.2)
Han ethnicity	1766 (47.6)	994 (60.6)^a^	569 (72.2)^a^
Number of household electrical appliances			
0–2	991 (26.7)	451 (27.5)	212 (26.9)
3	1282 (34.6)	636 (38.8)	278 (35.3)
4	1435 (38.7)	553 (33.7)^b^	298 (37.8)
Child having elder sibling(s)	1786 (48.2)	820 (50.0)	237 (30.1)^a^
Guardian’s education attainment^c^			
Illiteracy	627 (16.9)	328 (20.0)	345 (43.8)
Primary education	886 (23.9)	433 (26.4)	263 (33.4)
Junior high school	1,710 (46.1)	752 (45.9)	142 (18.0)
Senior high school and above	484 (13.1)	126 (7.7)^a^	38 (4.8)^a^
Stunting, height-for-age *Z* score <-2 standard deviation	609 (16.4)	247 (15.1)	132 (16.6)
Ever breastfed^d^	3489 (94.1)	1554 (94.8)	593 (75.3)^a^
Length of breastfeeding, months	9.8 (6.2)^e^	9.7 (5.9)^e^	7.2 (6.3)^e, a^
Early initiation of breastfeeding^d^	1,574 (42.5)	644 (39.3)^b^	263 (33.4)^a^
Age-appropriate breastfeeding^f^	1,014 (36.9)	514 (39.8)^b^	14 (3.0)^a^
Child complementary feeding^g^			
Minimum dietary diversity	2266 (69.0)	912 (63.0)^a^	543 (70.3)
Minimum meal frequency	1665 (50.7)	666 (46.0)^b^	257 (33.3)^a^
Minimum acceptable diet	973 (29.6)	377 (26.0)^b^	124 (16.0)^a^
Intake of iron-rich/fortified foods	2224 (67.7)	838 (57.9)^a^	492 (63.7)^b^
Milk feeding	2081 (56.1)	872 (53.2)^a^	574 (72.8)^a^

^a^ *p* < 0.001 compared to children with neither parent migrated

^b^ *p* < 0.05 compared to children with neither parent migrated

^c^2 children with missing information

^d^288 children with missing information

^e^Mean (standard deviation)

^f^Children aged 0–23 months (*N* = 4509 including 336 children with missing information)

^g^In children aged 6–35 months (*N* = 5505)

**Table 2:** Relative risks of stunting and feeding practices in children with father or mother migrated compared to children with neither parent migrated (*N* = 6136, China, 2010–2011)

Characteristics of children	Father migrated only		Mother with/without father migrated	
	*n* = 1640		*n* = 788	
	aOR	95 % CI	aOR	95 % CI
Stunting, height-for-age *Z* score <-2 standard deviation	0.93	0.75 to 1.15	0.78	0.65 to 0.95
Ever breastfed	0.99	0.56 to 1.75	0.30	0.17 to 0.52
Length of breastfeeding, months (*β* coefficient and 95 % CI)	0.02	–0.65 to 0.68	−3.77	−5.01 to −2.53
Early initiation of breastfeeding	0.83	0.60 to 1.14	0.83	0.54 to 1.27
Age-appropriate breastfeeding^a^	1.04	0.81 to 1.32	0.04	0.02 to 0.10
Child complementary feeding^b^				
Minimum dietary diversity	0.82	0.66 to 1.03	1.00	0.71 to 1.40
Minimum meal frequency	0.97	0.75 to 1.25	0.44	0.29 to 0.68
Minimum acceptable diet	0.98	0.81 to 1.19	0.56	0.39 to 0.79
Intake of iron-rich/fortified foods	0.74	0.63 to 0.88	0.94	0.67 to 1.33
Milk feeding	0.94	0.72 to 1.23	1.87	1.30 to 2.70

*aOR* odds ratio adjusted for child’s age, gender, ethnicity, whether the child had elder siblings, guardian’s education attainment, number of household electrical appliances and year of survey, *CI* confidence interval

^a^Children aged 0-23 months (*N* = 4509 including 336 children with missing information)

^b^In children aged 6-35 months (*N* = 5505)

**Fig. 1:**
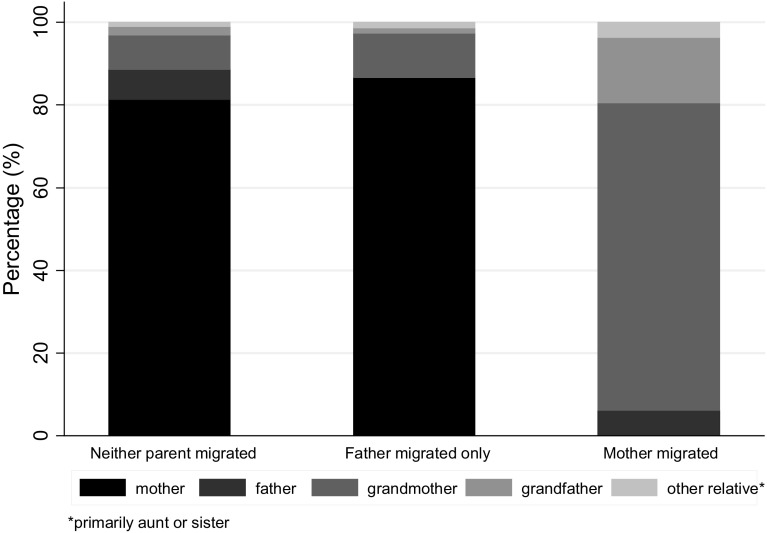
Primary guardian of child for left-behind and non-left-behind children (*N* = 6136, China, 2010–2011)

In terms of the guardianship, in non-left-behind children majority (81.3 %) were primarily cared by their mothers, and the proportion was similar in children with only the father migrated (86.7 %) (Fig. 1). However, when the mother migrated, the main guardian was most likely to be the grandmother (74.2 %) (Fig. 1). The prevalence of stunting was lowest (14.9 %) in children cared for by mothers followed by 18.4, 21.8, and 22.6 % in children whose primary guardian was a grandparent, father and other relative, respectively. Compared to children cared for by mothers, those with father or grandparent guardians had shorter breastfeeding periods and were less likely to receive age-appropriate breastfeeding and minimum acceptable diet (Tables 3, 4). Children cared for by a grandparent were in addition less likely to be ever breastfed (aOR = 0.29. 95 % CI 0.17–0.50) but more likely to have non-breast milk feeding (aOR = 2.44, 1.90–3.13) compared to children cared for by the mother (Table 4).

**Table 3:** Socio-demographic characteristics, stunting and feeding practices in children cared by different guardian (*N* = 6043, China, 2010–2011)

Characteristics of children	Mother	Father	Grandparent
	*n* = 4437	*n* = 316	*n* = 1290
	*n* (%)	*n* (%)	*n* (%)
Age of child, months			
0–5	551 (12.4)	24 (7.6)	52 (4.0)
6–11	1233 (27.8)	38 (12.0)	234 (18.1)
12–23	1620 (36.5)	141 (44.6)	557 (43.2)
24–35	1033 (23.3)	113 (35.8)^a^	447 (34.7)^a^
Child’s gender, male	2388 (53.8)	181 (57.3)	687 (53.3)
Han ethnicity	2376 (53.6)	163 (51.6)	749 (58.1)^b^
Number of household electrical appliances			
0–2	1124 (25.3)	119 (37.7)	377 (29.2)
3	1602 (36.1)	104 (32.9)	463 (35.9)
4	1711 (38.6)	93 (29.4)^a^	450 (34.9)^b^
Child having elder sibling(s)	2181 (49.2)	174 (55.1)^b^	448 (34.7)^a^
Guardian’s education attainment^c^			
Illiteracy	557 (12.6)	41 (13.0)	679 (52.6)
Primary education	1082 (24.4)	71 (22.5)	410 (31.8)
Junior high school	2254 (50.8)	151 (47.8)	162 (12.6)
Senior high school and above	543 (12.2)	53 (16.8)	38 (3.0)^a^
Stunting, height-for-age *Z* score <-2 standard deviation	661 (14.9)	69 (21.8)^b^	237 (18.4)^b^
Ever breastfed^d^	4303 (97.0)	276 (87.3)^a^	999 (77.4)^a^
Length of breastfeeding, months	10.2 (5.9)^e^	9.4 (6.6)^e, b^	7.1 (6.2)^e, a^
Early initiation of breastfeeding^d^	1909 (43.0)	122 (38.6)^a^	418 (32.4)^a^
Age-appropriate breastfeeding^f^	1387 (40.8)	44 (21.7)^a^	103 (12.2)^a^
Child complementary feeding			
Minimum dietary diversity	2630 (67.7)	206 (70.6)	829 (67.0)
Minimum meal frequency	1788 (46.0)	177 (60.6)^a^	573 (46.3)
Minimum acceptable diet	1085 (27.9)	93 (31.9)	271 (21.9)^a^
Intake of iron-rich/fortified foods	2533 (65.2)	195 (66.8)	768 (62.0)^b^
Milk feeding	2364 (53.3)	184 (58.2)	918 (71.2)^a^

93 children cared by other relative, e.g., aunt and sister were excluded from this table

^a^ *p* < 0.001 compared to children primarily cared by the mother

^b^ *p* < 0.05 compared to children primarily cared by the mother

^c^2 children with missing information

^d^257 children with missing information

^e^Mean (standard deviation)

^f^Children aged 0–23 months (*N* = 4450 including 332 children with missing information)

^g^In children aged 6–35 months (*N* = 5416)

**Table 4:** Adjusted odds ratios with 95 % CI for the association of socio-demographic characteristics, stunting and feeding practices with guardianship (*N* = 6043, China, 2010–2011)

Characteristics of children	Father		Grandparent	
	*n* = 316		*n* = 1290	
	aOR	95 % CI	aOR	95 % CI
Stunting, height-for-age *Z* score < -2 standard deviation	1.26	1.00 to 1.58	0.88	0.69 to 1.13
Ever breastfed	0.68	0.38 to 1.21	0.29	0.17 to 0.50
Length of breastfeeding, months (β coefficient and 95 % CI)	−1.63	−2.61 to −0.65	−4.23	−4.98 to −3.47
Early initiation of breastfeeding	1.06	0.86 to 1.31	0.76	0.52 to 1.12
Age-appropriate breastfeeding^a^	0.51	0.40 to 0.64	0.16	0.09 to 0.28
Child complementary feeding^b^				
Minimum dietary diversity	0.93	0.66 to 1.30	1.17	0.91 to 1.51
Minimum meal frequency	0.97	0.68 to 1.39	0.63	0.41 to 0.95
Minimum acceptable diet	0.74	0.58 to 0.94	0.69	0.51 to 0.93
Intake of iron-rich/fortified foods	0.87	0.68 to 1.12	1.09	0.85 to 1.40
Milk feeding	1.21	0.90 to 1.62	2.44	1.90 to 3.13

93 children cared by other relative, e.g., aunt and sister were excluded from this table

*aOR* odds ratio adjusted for child’s age, gender, ethnicity, whether the child had elder siblings, guardian’s education attainment, number of household electrical appliances and year of survey, *CI* confidence interval

^a^Children aged 0–23 months (*N* = 4509 including 336 children with missing information)

^b^In children aged 6–35 months (*N* = 5505)

The increased prevalence of stunting was not associated with children’s left-behind status, whether the child’s father or mother migrated (aORs = 0.94 and 0.79, 95 % CIs 0.77–1.16 and 0.61–1.03, respectively) (Table 5). Primary guardianship by the father, however, did show an association with stunting as children cared for by the father had a 60 % increased prevalence of stunting compared to children cared for by the mother (OR = 1.60, 1.26–2.01). This reduced slightly after adjustment for other important factors, yet the association remained statistically significant (aOR = 1.32, 95 % CI 1.04–1.67) (Table 5). Besides, the prevalence of stunting was also associated with age, gender, ethnicity, number of household electrical appliances and guardian’s education level (Table 5). In addition, the odds of stunting in children who had milk feeding was 22 % lower than the odds in children who had no milk feeding (aOR = 0.78, 0.62–0.98) (Table 5). The protective effect of milk feeding, however, was only for children aged 6 months and above (aOR = 0.75, 0.59–0.95) but not for children aged 0-5 months (aOR = 1.21, 0.76–1.94). Milk feeding was associated with better minimum dietary diversity and intake of iron-rick/fortified foods but poor minimum meal frequency. In the sub-group analysis of children aged 6–35 months where complementary feeding could be assessed, minimum dietary diversity, minimum meal frequency and intake of iron-rich/fortified foods showed no statistically significant associations with stunting (Table S2).

**Table 5:** The risk of stunting by children’s socio-demographic and parental factors (*N* = 6136, China, 2010–2011)

	Crude OR	95 % CI	Adjusted OR^a^	95 % CI
Children left behind				
Neither parent migrated	1.00		1.00	
Father migrated only	0.90	0.67 to 1.21	0.94	0.77 to 1.16
Mother with/without father migrated	1.02	0.83 to 1.27	0.79	0.61 to 1.03
Primary guardian				
Mother	1.00		1.00	
Father	1.60	1.26 to 2.01	1.32	1.04 to 1.67
Grandparent	1.29	1.00 to 1.66	1.05	0.79 to 1.39
Other relative	1.67	0.96 to 2.88	1.36	0.74 to 2.52
Age of child, months				
0–5	1.00		1.00	
6–11	0.85	0.54 to 1.33	1.08	0.68 to 1.72
12–23	1.95	1.28 to 2.97	2.10	1.35 to 3.26
24–35	2.56	1.64 to 4.02	2.67	1.71 to 4.18
Gender of child, male	1.33	1.19 to 1.49	1.36	1.19 to 1.56
Han ethnicity	0.61	0.46 to 0.81	0.80	0.65 to 0.99
Number of household electrical appliances				
0–2	1.00		1.00	
3	0.55	0.42 to 0.73	0.71	0.55 to 0.91
4	0.32	0.23 to 0.46	0.46	0.32 to 0.66
Child having elder sibling(s)	1.16	1.03 to 1.31	0.94	0.80 to 1.12
Guardian’s education level				
Illiteracy	1.00		1.00	
Primary education	0.71	0.57 to 0.87	0.83	0.68 to 1.03
Junior high school	0.45	0.33 to 0.60	0.63	0.44 to 0.89
Senior high school and above	0.37	0.28 to 0.49	0.56	0.40 to 0.78
Length of breastfeeding, months				
0–5	1.00		1.00	
6–11	0.77	0.55 to 1.07	0.85	0.68 to 1.07
12–35	1.55	1.09 to 2.22	1.12	0.88 to 1.42
Milk feeding	0.72	0.56 to 0.94	0.78	0.62 to 0.98

*OR* odds ratio, *CI* confidence interval

^a^Odds ratio mutually adjusted for the variables in the table as well as the year of survey

## Discussion

### Principal findings

Nearly 40 % of children in these rural areas of China were left behind by their parents. Among these children, about two-thirds of the children’s fathers migrated, leaving single mothers as principal guardians; whereas one-third of children’s mothers migrated, leaving guardianship primarily to grandmothers. Children with only father migrated had similar nutritional status to non-left-behind children. In contrast, children with mother migrated therefore, cared by non-mother guardian were less likely to receive age-appropriate breastfeeding and a minimum acceptable diet. Despite the difference in breastfeeding and child complementary feeding practices observed between mother and non-mother guardianship, our results suggested that the increased likelihood of stunting was only in children cared by father but not by grandparent. This might be related to an increase of milk feeding and subsequently better intake of iron-rich/fortified food and minimum dietary diversity in children having the grandparent as their main guardians.

### Strengths and limitations

Our study contains one of the largest samples of left-behind children thus far to examine the impact of both left-behind status and guardianship on the prevalence of stunting with consideration of feeding practices and basic socioeconomic factors which is commonly lacking in previous studies. Since stunting is mostly formed in children at very young age, our study population of children aged 0–3 years has enabled us to examine its likelihood in a crucial period of child development. The children’s anthropometric measurements (length and height) were measured by trained research assistants using standard measure tools. Although our study population has a very high stunting prevalence overall, this is similar to a recent estimate found in a separate study.(Chen et al. 2011) We were, however, unable to estimate the length of time for which children were left behind by their economic migrant parents due to lack of data in most children. However, in 695 children with such information, the median length of left-behind time was 8 months (interquartile range 4–15), indicating that our case definition is broadly comparable with the census definition.(National Bureau of Statistics of China 2012) Due to the nature of cross-sectional data, we were unable to assess causative factors for the high prevalence of stunting in our study population. Our presentation of how stunting prevalence varied by children’s socio-demographic and environmental factors should therefore, be interpreted cautiously in consideration of this.

### Interpretation in context of previous literature

Results from previous research have suggested that guardianship is an important factor to consider when examining the risk of stunting in left-behind children. Yu et al. including nearly 8,000 children under 5 years old obtained from surveillance data of rural areas of China found that there was a slightly increased risk of stunting in children not cared by their parents compared with those cared by parents (OR = 1.19, 95 % CI 1.02–1.39) (Yu et al. 2011). Another study using similar data with about 16,000 children aged under 5 years from 1990 to 2010 found that the prevalence of stunting in children left behind by migrant mothers was 20–30 % higher than in non-left-behind children in rural areas of China (Chen et al. 2011). Similar results of increased risks of stunting in very young children not primarily cared by mothers were also observed in other Chinese studies (He et al. 2007; Cui et al. 2008). Although in China it is quite common for children cared by their grandparents while one or both parents are absent, these studies have not examined the children’s dietary intake or growth taking into consideration of both their different left-behind status and their carers’ socioeconomic circumstances and health behaviours.

A case–control study including 774 children left behind by migrant mothers and 774 non-left-behind children found that left-behind children were less likely to be breastfed with shorter duration of breastfeeding and poor complementary feeding (Luo et al. 2008). However, this case–control study did not find statistically significant differences in the risk of stunting, between left-behind and non-left-behind children, (Luo et al. 2008) which is similar to our findings. This could be due to other health-related behaviours in caregivers such as non-breast milk feeding in left-behind children cared by non-mother guardians suggested in our study which has shown a statistically significant association with a lower prevalence of stunting.

### Conclusions and public health policy implications

Our study suggested that parent absence is not independently associated with stunting in children. Stunting is primarily associated with the child’s socio-demographic circumstances and the quality of care provided by the caregivers. In particular, single fathers need additional support on providing proper nutrition to their children. To reduce the stunting risk, policy needs to focus on promoting infant and child nutrition and providing infant and young child feeding counselling and support to caregivers in rural areas of China.

## Electronic supplementary material

Below is the link to the electronic supplementary material.

Supplementary material 1 (DOC 72 kb)
