# Supplementary material for: Child feeding and stunting prevalence in left-behind children: a descriptive analysis of data from a central and western Chinese population
Source: Int J Public Health. 2016 Jun 18;62(1):143–51. doi: 10.1007/s00038-016-0844-6 (PMC5288445; doi:10.1007/s00038-016-0844-6)
Supplement: Supplementary file 1 — Supplementary material 1 (DOC 72 kb) [file 38_2016_844_MOESM1_ESM.doc]

**Child feeding and stunting status in left-behind children: a descriptive analysis of data from a central and western Chinese population**

**International Journal of Public Health**

Lu Ban, Sufang Guo, Robert W. Scherpbier, Xiaoli Wang, Hong Zhou, Laila J. Tata

**Corresponding authors:**

Sufang Guo, UNICEF, [sguo@unicef.org](mailto:sguo@unicef.org)

Lu Ban, University of Nottingham, [lu.ban@nottingham.ac.uk](mailto:lu.ban@nottingham.ac.uk)

**Table** **S1** Characteristics of children, before and after weighting* (China, 2010-2011)

|  | **Un-weighted** | **Weighted** |
| --- | --- | --- |
|  | **N=6,136** | **N=6,189** |
|  | **n (%)** | **n (%)** |
| **Age of the child, months** |  |  |
| 0-5 | 631 (10.3) | 689 (11.1) |
| 6-11 | 1,516 (24.7) | 1,575 (25.5) |
| 12-23 | 2,362 (38.5) | 2,415 (39.0) |
| 24-35 | 1,627 (26.5) | 1,510 (24.4) |
| **Child’s gender** |  |  |
| Male | 3,306 (53.9) | 3,412 (55.1) |
| Female | 2,830 (46.1) | 2,777 (44.9) |
| **Ethnicity** |  |  |
| Han | 3,329 (54.3) | 3,695 (59.7) |
| Others | 2,807 (45.8) | 2,494 (40.3) |
| **Number of household electrical appliances** |  |  |
| 0-2 | 1,654 (27.0) | 1,621 (26.2) |
| 3 | 2,196 (35.8) | 2,201 (35.6) |
| 4 | 2,286 (37.3) | 2,367 (38.3) |
| **Children left-behind status** |  |  |
| None (both parents around) | 3,708 (60.4) | 3,704 (59.9) |
| Father migrated only (mother around) | 1,640 (26.7) | 1,638 (26.5) |
| Mother migrated with or without father migrated | 788 (12.8) | 847 (13.7) |
| **Primary guardian** |  |  |
| Mother | 4,437 (72.3) | 4,537 (73.3) |
| Father | 316 (5.2) | 315 (5.1) |
| Grandmother | 1,071 (17.5) | 1,020 (16.5) |
| Grandfather | 219 (3.6) | 226 (3.7) |
| Other relative (e.g. aunt or sister) | 93 (1.5) | 91 (1.5) |

*adjustment for unequal probabilities of selection of children under three years old in different rural counties in China

**Table S2 The risk of stunting by children’s socio-demographic and parental factors in children aged 6-35 month old (N=5,505, China, 2010-2011)**

|  | **Crude OR** | **95%CI** | **Adjusted OR^a^** | **95%CI** |
| --- | --- | --- | --- | --- |
| **Children left behind** |  |  |  |  |
| Neither parent migrated | 1.00 |  | 1.00 |  |
| Father migrated only | 0.90 | 0.68-1.19 | 0.94 | 0.77-1.13 |
| Mother with/without father migrated | 0.97 | 0.79-1.19 | 0.77 | 0.59-1.01 |
| **Primary guardian** |  |  |  |  |
| Mother | 1.00 |  | 1.00 |  |
| Father | 1.61 | 1.28-2.03 | 1.37 | 1.08-1.73 |
| Grandparent | 1.24 | 0.96-1.59 | 1.08 | 0.81-1.44 |
| Other relative | 1.67 | 0.97-2.87 | 1.41 | 0.78-2.57 |
| **Age of child, months** |  |  |  |  |
| 6-11 | 1.00 |  | 1.00 |  |
| 12-23 | 2.29 | 1.76-2.97 | 2.08 | 1.52-2.84 |
| 24-35 | 3.01 | 2.19-4.14 | 2.66 | 1.98-3.59 |
| **Gender of child, male** | 1.33 | 1.19-1.50 | 1.34 | 1.18-1.53 |
| **Han ethnicity** | 0.60 | 0.45-0.79 | 0.81 | 0.68-0.98 |
| **Number of household electrical appliances** |  |  |  |  |
| 0-2 | 1.00 |  | 1.00 |  |
| 3 | 0.53 | 0.40-0.70 | 0.69 | 0.53-0.88 |
| 4 | 0.32 | 0.22-0.46 | 0.46 | 0.33-0.65 |
| **Child having elder sibling(s)** | 1.17 | 1.03-1.34 | 0.94 | 0.80-1.11 |
| **Guardian’s education level** |  |  |  |  |
| Illiteracy | 1.00 |  | 1.00 |  |
| Primary education | 0.75 | 0.61-0.93 | 0.91 | 0.75-1.11 |
| Junior high school | 0.46 | 0.34-0.60 | 0.66 | 0.47-0.91 |
| Senior high school and above | 0.39 | 0.29-0.52 | 0.62 | 0.45-0.85 |
| **Length of breastfeeding, months** |  |  |  |  |
| 0-5 | 1.00 |  | 1.00 |  |
| 6-11 | 0.62 | 0.46-0.85 | 0.87 | 0.69-1.10 |
| 12-35 | 1.26 | 0.91-1.73 | 1.11 | 0.86-1.45 |
| **Minimum dietary diversity** | 0.78 | 0.61-0.99 | 0.81 | 0.65-1.02 |
| **Minimum meal frequency** | 1.21 | 0.80-1.82 | 0.93 | 0.76-1.14 |
| **Intake of iron-rich/fortified foods** | 0.93 | 0.71-1.21 | 0.98 | 0.78-1.24 |
| **Milk feeding** | 0.64 | 0.50-0.84 | 0.78 | 0.62-0.98 |

^a^ odds ratio mutually adjusted for the variables in the table as well as the year of survey;

OR=odds ratio;

95%CI=95% confidence interval
